# *In vivo* and *in vitro* effects of crocetin and its amide derivative on acrylamide-induced neurotoxicity

**DOI:** 10.22038/AJP.2023.22316

**Published:** 2024

**Authors:** Amir Hossein Ajzashokouhi, Bibi Marjan Razavi, Hamid Sadeghian, Hossein Hosseinzadeh

**Affiliations:** 1 *School of Pharmacy, Mashhad University of Medical Sciences, Mashhad, Iran*; 2 *Student Research Committee, Mashhad University of Medical Sciences, Mashhad, Iran*; 3 *Targeted Drug Delivery Research Center, Pharmaceutical Technology Institute, Mashhad University of Medical Sciences, Mashhad, Iran*; 4 *Department of Pharmacodynamics and Toxicology, School of Pharmacy, Mashhad University of Medical Sciences, Mashhad, Iran*; 5 *Department of Laboratory Sciences, School of Paramedical Sciences, Mashhad University of Medical Sciences, Mashhad, Iran*; 6 *Pharmaceutical Research Center, Pharmaceutical Technology Institute, Mashhad University of Medical Sciences, Mashhad, Iran*

**Keywords:** Neurotoxicity, Trans-sodium crocetinate, Bis-N-(N-methylpyprazinyl) crocetinate, Acrylamide, Saffron, Oxidative stress

## Abstract

**Objective:**

Acrylamide (ACR) is a neurotoxic agent whose damage could be attenuated by antioxidants administration. Crocetin is a saffron-derived antioxidant that has neuroprotective effects. This study evaluates the protective effects of trans-sodium crocetinate (TSC) and its water-soluble derivative, Bis-N-(N-methylpyprazinyl) crocetinate (BMPC) against ACR neurotoxicity.

**Materials and Methods:**

PC12 cells were treated with TSC and BMPC (1.95, 3.9, 7.81, 15.62, 31.25, 62.5, 125, 250, 500, and 1000 μM) for 24 hr. ACR was then added at a concentration of 6.5 mM (IC_50_), and cell viability was assessed by 3-(4,5-dimethylthiazol-2-yl)-2, 5-diphenyltetrazolium bromide. In the *in vivo* study, male Wistar rats were treated with ACR (50 mg/kg, intraperitoneal (i.p.)) for 11 days alone or in combination with TSC and BMPC (2.5, 5, and 10 mg/kg, i.p.) or vitamin E (200 IU/kg, i.p.). Motor impairments were then evaluated. The cerebral cortex of sacrificed rats was taken for the malondialdehyde (MDA) and glutathione (GSH) levels measurement.

**Results:**

*In vitro* studies showed that TSC at a concentration of 7.81 μM and BMPC at concentrations of 3.9, 7.81, and 15.62 μM exhibited the lowest toxicity in acrylamide administration. In the *in vivo* study, pretreatment with 2.5, 5, and 10 mg/kg of TSC ameliorated behavioral impairments, but BMPC could not attenuate them. GSH and MDA were improved by 2.5, 5, and 10 mg/kg TSC and 2.5 mg/kg BMPC.

**Conclusion:**

TSC and BMPC administration improved behavioral index and oxidative stress injuries in Wistar rats exposed to ACR through MDA reduction and GSH content enhancement in the cerebral cortex.

## Introduction

Acrylamide (ACR) is a water-soluble, low molecular weight toxic compound that is formed as an intermediate product of the Millard reaction during the heating process (Mottram et al., 2002). ACR is known as a neurotoxin and carcinogenic compound produced when carbohydrate-rich foods are cooked and fried at temperatures above 120°C (Tareke et al., 2000; Mehri et al., 2012). Also, it is used in many industries, such as the polymer industry and wastewater treatment (Emmons and Stevens, 1983). Fortunately, scientists believe that exposure to low doses of this toxin from foods may not cause acute toxicity, but many neurologists are concerned about its cumulative effects (LoPachin, 2004; Motamedshariaty et al., 2014). The neurotoxic effects of ACR include muscle weakness, weight loss, walking difficulty, Parkinsonism, and ataxia (Moser et al., 1992; Shell et al., 1992; LoPachin et al., 2002). Several mechanisms cause ACR neurotoxicity, one of which is oxidative stress induction (Mehri et al., 2014; Mehri et al., 2015). Therefore, it seems that using antioxidant agents can reduce the toxicity of this compound (Mohammadzadeh et al., 2018). 

Crocetin, a carotenoid dicarboxylic acid, is one of the active ingredients in saffron that has numerous pharmacological effects, including antioxidant and neuroprotective effects (Ahmad et al., 2005). Its beneficial effects include reducing fatigue and depression, and improving retinopathy, and sleep disorders were shown (Mizuma et al., 2009; Kuratsune et al., 2010; Yamauchi et al., 2011). Moreover, crocetin prevented 6-hydroxydopamine-induced degeneration of nigrostriatal dopaminergic neurons and maintained dopamine levels close to basal levels (Ahmad et al., 2005). In addition, it could increase oxygen permeability to the body fluids and plasma; thereby, this compound could improve alveolar filtration, increase oxygen delivery to the brain, and finally reduced damage to the nervous system (Giaccio, 2004). Other effects of this compound include anti-tumor, antidiabetics, anti-inflammatory, anti-hyperlipidemia, hepatoprotective, cardioprotective, and fertilization boosting (Hashemi and Hosseinzadeh, 2019). 

**Figure 1:**
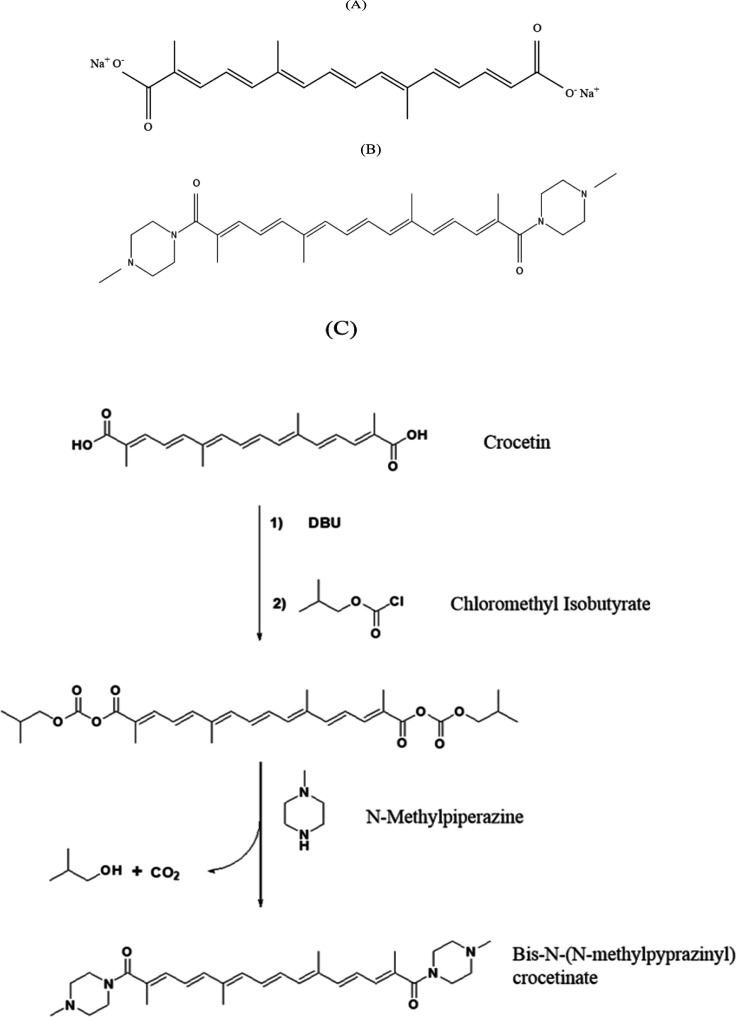
Trans-sodium crocetinate (A), Bis-N- (N- methylpyprazinyl) crocetinate (B), and the production process of Bis-N- (N- methylpyprazinyl) crocetinate (C).

Crocetin is not water-soluble at the physiologic pH (Hashemi and Hosseinzadeh, 2019), so in this study, at first, the various salts of crocetin were synthesized, and their radical hunting power was measured. Finally, the derivatives with the highest level of radical hunting power and water solubility were chosen. The neuroprotective effects of sodium and amid derivatives of crocetin, including Trans-sodium crocetinate (TSC, Figure 1A) and Bis-N-(N-methylpyprazinyl) crocetinate (BMPC, Figure1B), against ACR toxicity in rats and PC12 cells were investigated. 

## Materials and Methods

**Chemicals**

RPMI 1640 Medium and Fetal Bovine Serum (FBS) were obtained from Gibco. Also, (4, 5- dimethylthiazol-2-yl)-2, 5-diphenyl tetrazolium (MTT), TBA (2-thiobarbituric acid), phosphoric acid, potassium chloride, ACR, and n-butanol were purchased from Merck. The injectable form of vitamin E was purchased from Osveh Company, Iran. Trans-sodium crocetinate (TSC) was purchased from Tinabshimi Company, Iran. Bis-N-(N-methylpyprazinyl) crocetinate (BMPC) was synthesized by ourselves. 

**Synthesis of Bis-N- (N- methylpyprazinyl) crocetinate (novel compound)**

Isobutyl chloroformate (3.0 g, 22 mmol) was added dropwise to a solution of crocetin (3.3 g; 10 mmol) and 3.5 g of 1,8-Diazabicyclo [5.4.0] undec-7-ene (DBU) in 60 ml dried chloroform while stirring in ice and water mixture.

After removing the ice bath, the reaction mixture was stirred at room temperature for two hr. N-methylpiperazine (2 g; 20 mmol) was added dropwise and stirring was completed after three hours. Sediments were separated, washed twice with water (2 × 50 ml), and dried. Finally, the desired product (Bis (4-methylpiperazin-1-yl) crocetin)) was obtained as a deep violet powder.

**Cell culture**

PC12 cells were purchased from the Pasteur Institute (Tehran, Iran). Cells were kept at 37°C in a humidified atmosphere (90%) containing 5% of CO_2_. Cells were cultured in Roswell Park Memorial Institute (RPMI) 1640 medium supplemented with 10% (v/v) fetal bovine serum, 100 U/ml penicillin, and 100 μg /ml streptomycin.

**Cell viability**

Cultured cells viability was assessed by MTT assay (Freimoser, Jakob et al. 1999). In a 96-well microliter plate of PC12 cells at a density of 5000 cells/well, in the first stage, 24- and 48-hr toxicity of TSC and BMPC was evaluated, and concentrations with the lowest toxicity were selected. Next, we calculated the 24-hr toxicity of ACR and its IC_50_ value. Finally, after pretreatment with TSC and BMPC (1.25, 3.9, 7.81, and 15.62 μM) for 24 hr, ACR at a concentration of 6.5 mM in phosphate-buffered saline (PBS) was added to each well. After keeping the cells in an incubator for 24 hr, they were treated with MTT solution (0.5 mg/ml PBS) at 37°C for 3 hr. Dimethyl sulfoxide (DMSO) was used to dissolve formazan crystals, and ELISA reader (Start Fax-2100, UK) metered absorbance of the solution at 545 nm (630 nm as reference) (Tandisehpanah et al., 2022). 

**Experimental animals**

Seventy-two male Wistar rats (200–250 g) were housed in colony rooms with a 12/12 hours light/dark cycle at 21 ± 2°C with free access to food and water. Mashhad University of Medical Sciences Ethics committee approved the present study (IR.MUMS.SP.1395.52).

**Experimental design**

Neurotoxicity was induced by intraperitoneal (i.p.) injection of ACR (50 mg/kg/day) in Wistar rats for 11 days (LoPachin, 2005). The aforementioned doses and routes of administration are well-distinguished by neuropathological manifestations and neurological impairments. In this experiment, ACR, TSC, and BMPC were dissolved in normal saline, and rats were separated into 12 random groups (n = 6 rats for each group) and treated as follows: 

1) Control, normal saline. 

2) ACR (50 mg/kg, i.p.) for 11 days. 

3) ACR (50 mg/kg, i.p.) for 11 days + Vitamin E (200 mg/kg, i.p.). 

4) ACR (50 mg/kg, i.p.) for 11 days + TSC (2.5 mg/kg, i.p.). 

5) ACR (50 mg/kg, i.p.) for 11 days + TSC (5 mg/kg, i.p.). 

6) ACR (50 mg/kg, i.p.) for 11 days + TSC (10 mg/kg, i.p.). 

7) TSC (10 mg/kg, IP) for 11 days. 

8) ACR (50 mg/kg, i.p.) for 11 days + BMPC (1.25 mg/kg, i.p.). 

9) ACR (50 mg/kg, i.p.) for 11 days + BMPC (2.5 mg/kg, i.p.). 

10) ACR (50 mg/kg, i.p.) for 11 days + BMPC (5 mg/kg, i.p.). 

11) ACR (50 mg/kg, i.p.) for 11 days + BMPC (10 mg/kg, i.p.). 

and 12) BMPC (10 mg/kg, i.p.) for 11 days.

**The behavioral index (gait scores) evaluation**

After the treatment of the animals, the gait scores were determined based on the LoPachin methods (LoPachin, 2005). The rats were placed in a clear Plexiglas box and observed for 3 min. Based on observation, a gait score (1 to 4) was given. These scores include 1 = a normal, unaffected gait; 2 = a slightly impressed gait (foot splay, slight hind limb weakness, and spread); 3 = a moderately impaired gait (foot splay, moderate hind limb weakness, mild limb spread during ambulation) and 4 = a severe gait disturbance (foot splay, severe hind limb weakness, dragging hind limbs, and inability to rear).

**Tissue sampling**

After the gait score determination, rats were sacrificed under ketamine: xylazine anesthesia [80-100 mg/kg of ketamine in combination with 5-10 mg/kg of xylazine, i.p.], and their brain cortex was dissected for biochemical tests. The samples were snap-frozen in liquid nitrogen and stored at −80°C fridge until use.

**Biochemical assay**

Lipid peroxidation was evaluated by measuring thiobarbituric acid reactive products (TBAR) in rat cerebral cortical tissue. Malondialdehyde (MDA) as the final product of lipid peroxidation forms a red complex in combination with TBA with maximum absorbance at 532 nm. MDA was measured as a marker of lipid peroxidation. The brain cortex was homogenized using cold KCl solution (1.1 5%) to make a homogenous suspension (10%). Then, 0.5 ml of the suspension was poured into a 10- ml tube, then 3 ml of phosphoric acid (1%) and 1 ml of TBA were added. The tube was then placed in a boiling water bath for 45 min. The mixture was then cooled, 4 ml of n-butanol was poured into the mixture, and the tube was vortexed for about one minute. The mixture was centrifuged (3000 g, for 20 min) to separate the red-colored upper phase. Finally, the absorbance was measured using a spectrophotometer. The MDA standard curve (0–100 μM) was prepared (Tabeshpour et al., 2020).

The GSH content was determined following the reductive cleavage of 5, 5'-dithio-bis-(2-nitrobenzoic acid) by sulfhydryl groups to form a yellow color. To prepare a tissue homogenate of 10% in PBS, the brain cortex tissues was added to phosphate-buffered saline (0.1 M, pH 7.4). Next, 0.5 ml of tissue homogenate was mixed with 0.5 ml of 10% TCA (trichloroacetic acid). The mixture was vortexed and centrifuged at 3000 g for 10 min. The supernatant was obtained, and 2.5 ml of phosphate-buffered saline (pH 8) and 0.5 ml of DTNB were added. The absorbance was determined at 412 nm by a spectrophotometer (Jenway 6105 UV/Vis, UK). The GSH standard curve was prepared, and the results were illustrated as nmol/g tissue (Moron et al., 1979).

**Statistical analysis**

Data are presented as mean±S.D. Statistical analyses were performed using one-way ANOVA, followed by the Tukey-Kramer test to compare differences between means. For the gait scores, data are displayed as median with an interquartile range for each group, and statistical analysis was done using the nonparametric test Kruskal–Wallis. Differences at p<0.05 were considered statistically significant.

## Results

**Effects of ACR, TSC, and BMPC on PC12 cells**

**Figure 2:**
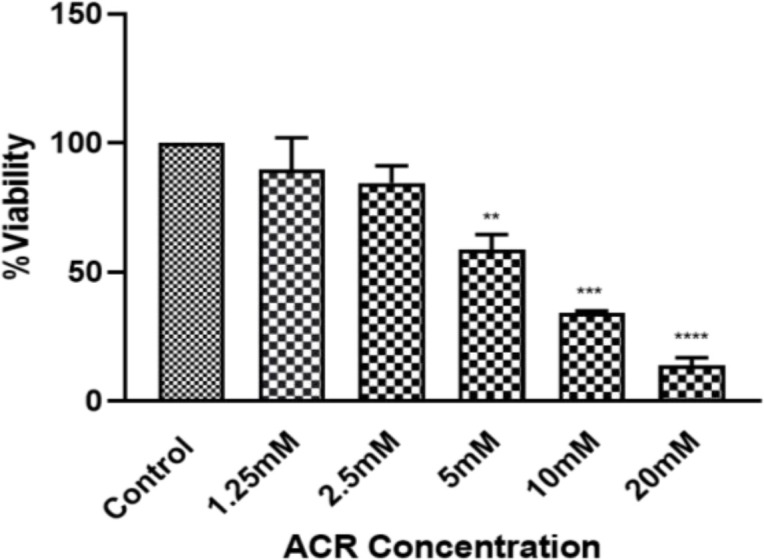
The effect of ACR on PC12 cell viability. Cells were exposed to various concentrations of ACR for 24 hr. Data are presented as Mean±SD from 3 separate plates (n=3). One-way ANOVA followed by the Tukey-Kramer test was used for statistical analysis. **p<0.01, ***p<0.001, and ****p<0.0001 compared to control. The IC50 calculated for ACR was 6.5±0.2 mM.

**Figure 3:**
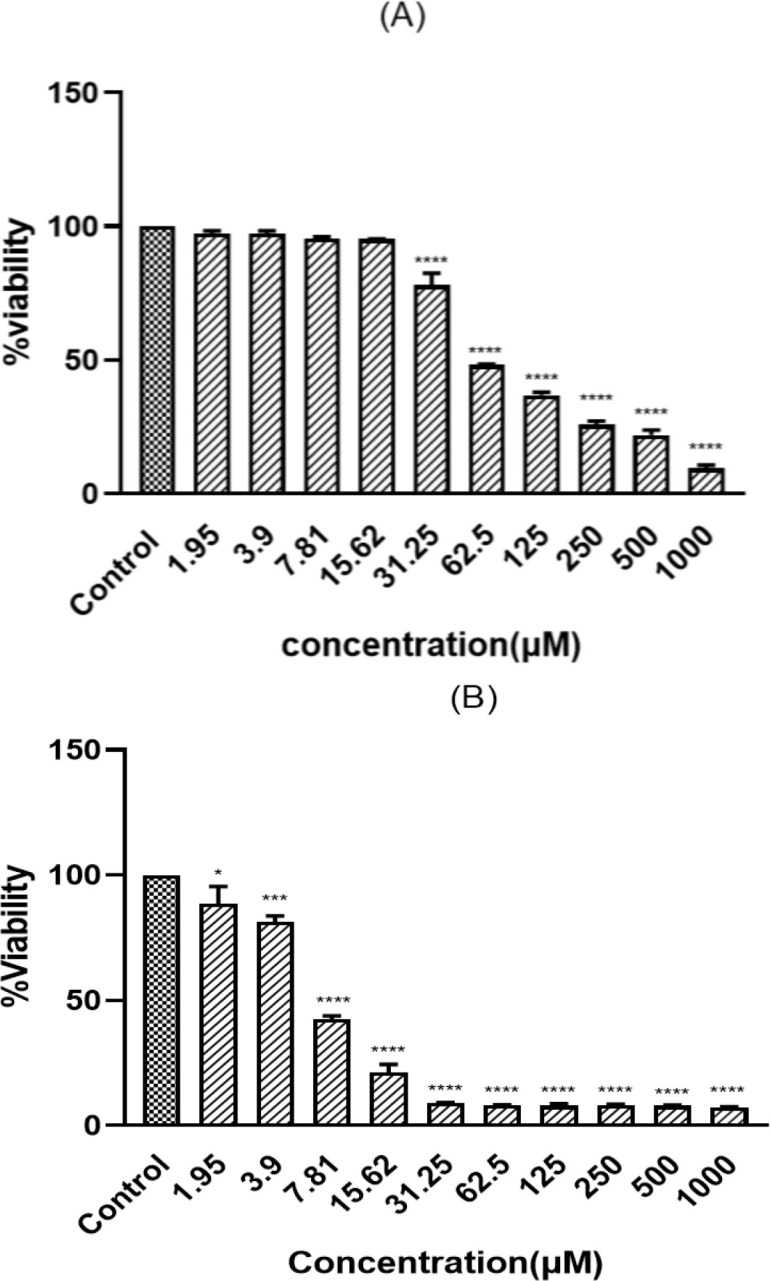
Effect of TSC on PC12 cell viability. Cells were incubated for 24 (A) and 48 hr (B) with different concentrations of TSC. Data are expressed as mean±SD of three separate experiments (n=3). One-way ANOVA followed by the Tukey-Kramer test was used for statistical analysis. (A) ****p<0.0001 and (B) *p<0.05, ***p<0.001, and ****p<0.0001 compared to control.

**Figure 4:**
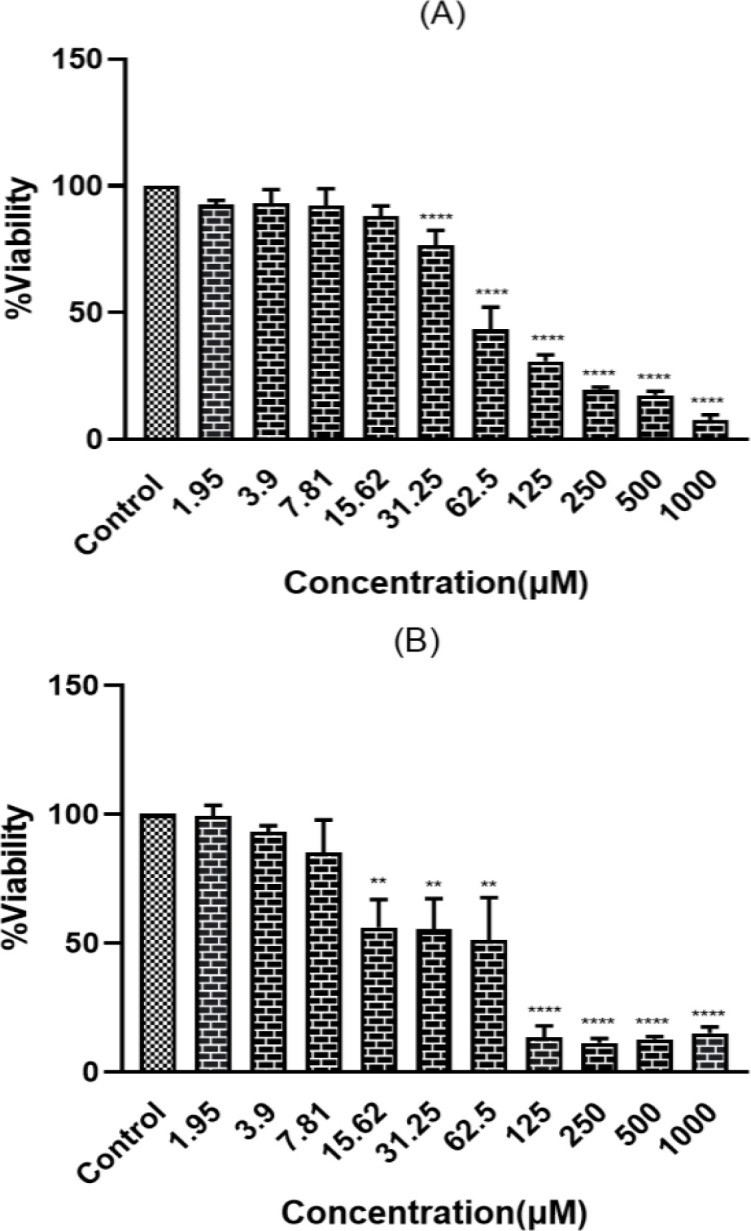
Effect of BMPC on PC12 cell viability. Cells were incubated for 24 (A) and 48 hr (B) with different concentrations of BMPC. Data are expressed as mean±SD of three separate experiments (n=3). One-way ANOVA followed by the Tukey-Kramer test was used for statistical analysis. (A) ****p<0.0001, (B) **p<0.01, and ****p<0.0001 compared to control.

**Figure 5:**
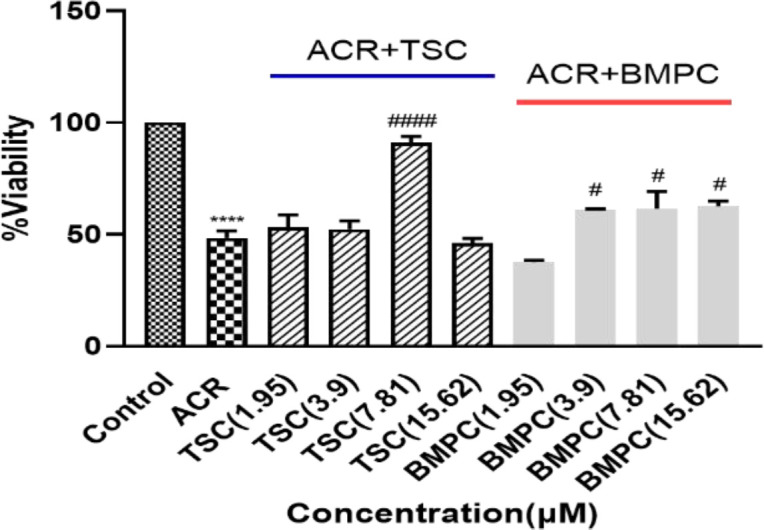
Effect of TSC and BMPC on ACR-induced cytotoxicity on PC12 cells. Cells were exposed to different concentrations of TSC for 24 hr and then exposed to TSC and ACR for 24 hr. Data are presented as Mean±SD, (n=3). One-way ANOVA followed by the Tukey-Kramer test was used for statistical analysis. #p<0.05 and ####p<0.0001 compared to ACR group and ****p<0.0001 compared to control group.

PC12 cells were exposed to different concentrations of ACR (1.25 mM to 20 mM) for 24 hr, and their viability was measured by MTT assay. ACR reduced cell viability in a concentration-dependent manner (Figure 2). The IC50 calculated for ACR was 6.5±0.2 mM. To determine the nontoxic concentrations of TSC and BMPC, we added them to cells at different concentrations for 24 and 48 hr (1.95, 3.9, 7.81, 15.62, 31.25, 62.5, 125, 250, 500, and 1000 μM). The BMPC production process is described in Figure 1C. Exposure to high concentrations (greater than 15.62 µM) of TSC and BMPC for 24 hr significantly decreased cell viability (Figures 3A and 4A, p<0.0001). On the other hand, in 48-hr incubation, TSC caused toxicity, and the nontoxic concentrations for BMPC was less than 15.61 µM (Figures 3B and 4B). Then, cells were pretreated with various concentrations of TSC and BMPC (1.95, 3.9, 7.81, and 15.62 μM) for 24 hr, and after it, ACR (6.5 mM) was added to each well. Pretreatment by TSC and BMPC for 24 hr significantly attenuated ACR cytotoxicity at 7.81 μM of TSC (p<0.0001) and 3.9, 7.81, and 15.62 μM of BMPC (p<0.05, Figure 5).

**Effects of ACR, TSC, and BMPC on the behavioral index (gait scores)**

**Figure 6:**
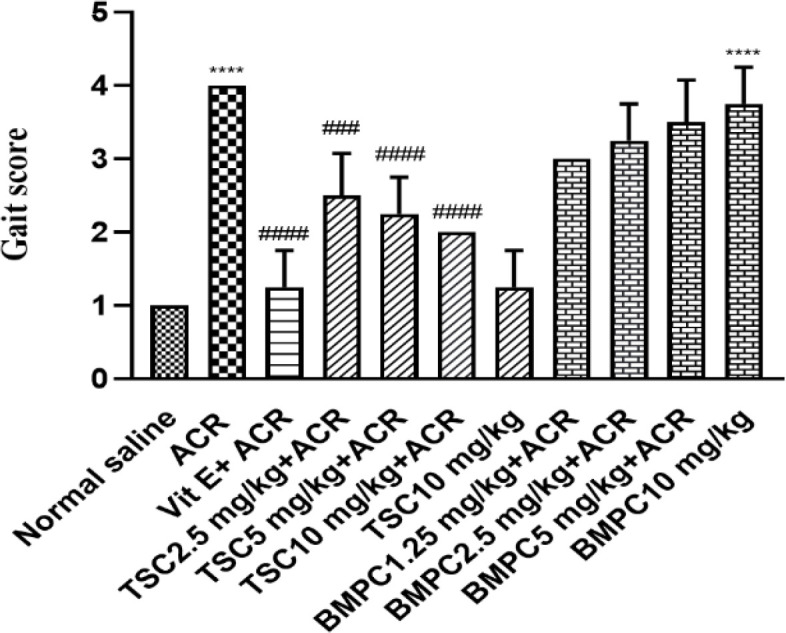
Effect of TSC and BMPC on ACR-induced movement disorder. Male Wistar rats were pretreated for 3 days with TSC (2.5, 5, and 10 mg/kg), BMPC (1.25, 2.5, and 5 mg/kg), and vitamin E (200 IU/kg) as a positive control (i.p.). Then for 11 days, TSC and BMPC (every day) and vitamin E (every other day) at the mentioned doses were injected, and after 30 min, 50 mg/kg of ACR was injected (i.p.) (every day). Bars represent the median with an interquartile range, (n = 6). The nonparametric Kruskal–Wallis test performed the statistical analysis. ****p<0.0001 compared to control group, ###p<0.001 and ####p<0.0001 compared to ACR group.

ACR exposure (50 mg/kg, i.p.) for 11 days led to a significant increase in the scoring index in comparison with the control group (p<0.0001). This index significantly decreased in animals that received vitamin E or 2.5, 5, and 10 mg/kg of TSC (p<0.001). The group that received BMPC at all doses (1.25, 2.5, 5, and 10 mg/kg) did not show any significant change compared to the group that received ACR alone. Movement disorders observed in the ACR group included foot splay, inability to use the hind limbs, and tremors (Figure 6).

**Effects of ACR, TSC, and BMPC on GSH content in the cerebral cortex**

**Figure 7:**
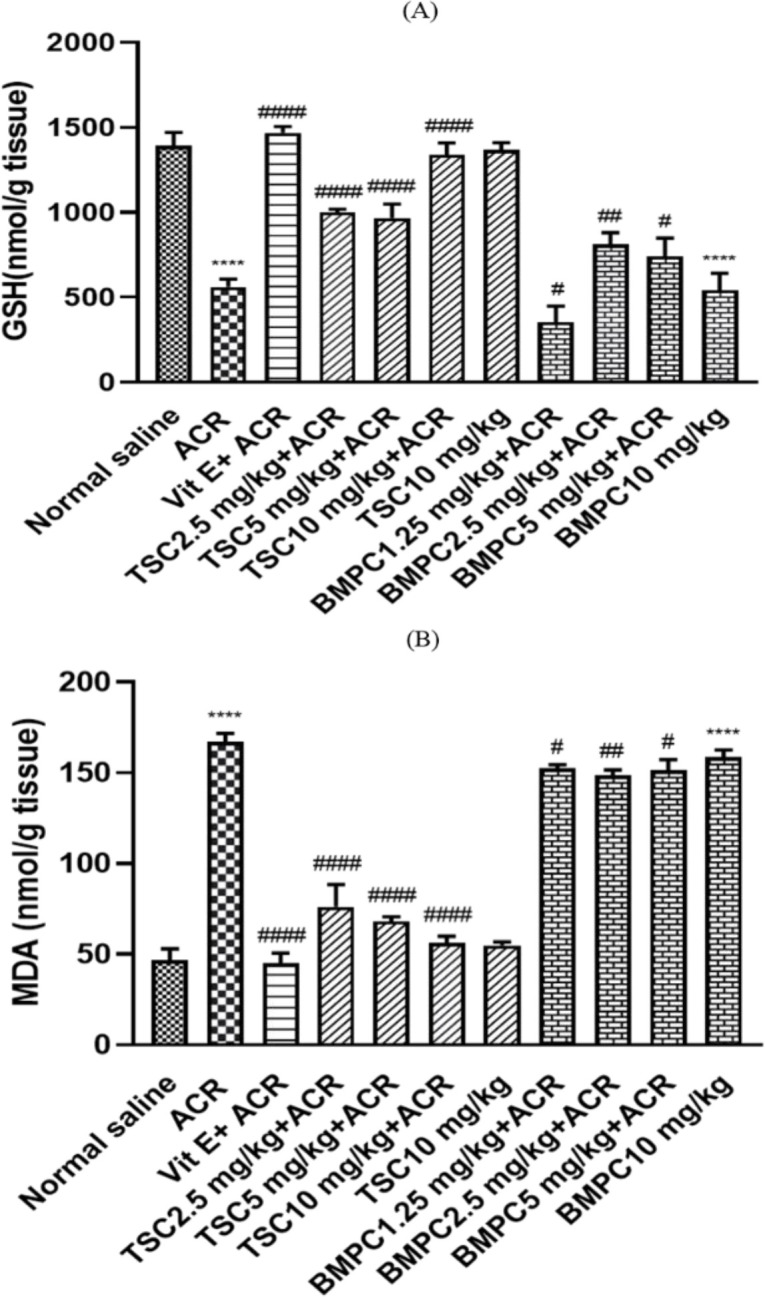
The effect of TSC and BMPC on ACR-induced glutathione content (A) and malondialdehyde (B) in the cerebral cortex. Male Wistar rats were pretreated for 3 days with TSC (2.5, 5, and 10 mg/kg), BMPC (1.25, 2.5, and 5 mg/kg), and vitamin E (200 IU/kg) as a positive control (i.p.). Then for 11 days, TSC and BMPC (every day) and vitamin E (every other day) at the mentioned doses, and after 30 min, 50 mg/kg of ACR was injected (every day, i.p.). Data are expressed as mean±SD (n=6). One-way ANOVA followed by the Tukey-Kramer test was used for statistical analysis. (A) #p<0.05, ##p<0.01, and ####p<0.0001 compared to ACR group and ****P<0.0001 compared to control group, (B) #p<0.05, ##p<0.01 and ####p<0.0001 compared to ACR group and ****p<0.0001 compared to control group.

After behavioral index measuring, the absorbance of the cerebral cortex obtained from sacrificed rats was evaluated at 412 nm. The data were plotted on a standard glutathione diagram, and the glutathione content was calculated. ACR administration significantly reduced GSH levels in the cortex in comparison with the control group (p<0.0001). Vitamin E (200 IU/ kg), TSC (2.5, 5, and 10 µM, p<0.0001), and BMPC (1.25, 2.5, and 5 µM, p<0.05) significantly elevated the GSH level in the cerebral cortex in comparison with ACR-treated rats group (Figure 7A). 

**Effects of ACR, TSC, and BMPC on MDA level in the cerebral cortex**

Lipid peroxidation in the brain cortex was also metered. Rats exposed to ACR showed a significant soar in MDA levels in comparison with the control group (p<0.0001). TSC (2.5, 5, and 10 mg/kg, p<0.0001), BMPC (1.25, 2.5, and 5 mg/kg, p<0.05), and vitamin E (200 IU/kg) treatment significantly reduced the level of MDA in the brain cortex in comparison with the ACR-only group (Figure 7B).

## Discussion

ACR is a hydrophilic vinyl monomer widely used in various industries (Greenberg et al., 2020). It is also found in high-carbohydrate foods that are cooked at high temperatures. The mechanism of ACR neurotoxicity is not fully understood. Nevertheless, studies have shown various mechanisms that damage the central and peripheral nervous system by inducing oxidative stress and apoptosis leading to weight loss, skeletal muscle weakness, and ataxia. *In vitro* studies have shown that ACR in PC12 cells causes significant toxicity in a time and concentration dependent manner (Esmaeelpanah et al., 2018), so it is vital to study the toxicity of this compound and possible protective compounds (Shipp et al., 2006). ACR toxicity appears to be reduced through the use of antioxidants. Crocetin, an active ingredient in saffron, has several pharmacological effects, including antioxidant (Jagadeeswaran et al., 2000) and neuroprotective (Giaccio, 2004) effects. Therefore, in this study, we investigated the protective effects of TSCs and BMPCs against ACR-induced neurotoxicity.

In the *in vitro *study, TSC at a concentration of 7.81 μM and BMPC at a concentration of 3.9, 7.81, and 15.62 μM after 24 hr showed the best effect in inhibiting cell toxicity caused by ACR. Crocetin derivatives are antioxidants that reduce oxidative stress by reducing ROS production (Magesh et al., 2006). The results of this study appear to be due to ACR-induced oxidative stress attenuation on PC12 cells by the high antioxidant capacity of crocetin. Also, investigations have shown that crocetin has cellular protective properties. Although the mechanisms of ACR neurotoxicity are undetermined, it seems that apoptosis and oxidative stress are involved in peripheral and central nervous system toxicity through the activation of caspase-3 and increased sub-G1 population of SH-SY5Y cells (Sumizawa and Igisu, 2007).

Furthermore, ACR induced apoptosis in PC12 cells through the bax/bcl2 ratio increasing and activating caspase 3. This is involved in increased ROS production (Esmaeelpanah et al., 2018). Crocetin showed some anti-apoptosis properties, but no specific mechanism has been discovered yet (Xiang et al., 2006). We, therefore, hypothesize that the results obtained in this study should be due to the anti-apoptotic effect of crocetin, though more studies are needed to understand the exact mechanism of this effect. Some researchers believe that crocetin's protective effects are related to its anti-inflammatory effects. Crocetin has anti-inflammatory effects by suppressing nitric oxide (NO), interleukin 1 beta (IL-1β), tumor necrosis alpha (TNF-α), reactive oxygen species (ROS) (Yang et al., 2006, Tamaddonfard et al., 2013). One study investigated the protective effect of crocetin on cell death due to beta-amyloid in HT22 hippocampal cells. The presence of beta-amyloid is one of the obvious signs of Alzheimer's disease (Kong et al., 2014). Another investigation has revealed that crocetin at concentrations of 1-10 μM can significantly reduce cell death and the ROS generation induced by beta-amyloid peptides (Yoshino et al., 2014). A similar study showed that crocetin not only decreased the production of beta-amyloid-activated oxygen species but also increased the mitochondrial membrane potential of cells and activated extracellular signal-regulated kinase 1/2 phosphorylation (Kong et al., 2014). Another study on crocetin's effect in inhibiting synaptic transmission caused by glutamate was performed on rat cerebral cortex cells in the physiological organs bath. In this study, glutamate with a concentration of 500 μM was added to the organ bath, which depolarized the synaptic membrane, and crocetin with a concentration range of 1-50 μM was able to inhibit glutamate-induced synaptic depolarization (Berger et al., 2011).

About our study, there are some important points in interpreting test results. Preliminary studies of the protective material of this study showed that in 24-hr incubation of PC12 cells, TSC showed less toxicity than BMPC. Still, in 48 hr of incubation, the toxicity of TSC sharply increased, while BMPC became slightly more toxic. So, the toxicity of TSC is time-dependent, while the toxicity of BMPC depends less on time. In the *in vivo *study, pretreatment with doses of 2.5, 5, and 10 mg/kg of TSC and 200 IU/kg of vitamin E significantly inhibited motor disorders, but BMPC could not attenuate movement disorders. ACR administration caused glutathione content to decrease and lipid peroxidation to increase, which is significantly inhibited by TSC (2.5, 5, and 10 mg/kg), BMPC (2.5 mg/kg), and vitamin E (200 IU/kg). Other studies show the use of antioxidant natural agents to reduce the neurotoxicity caused by ACR. One of these studies is about ginseng. This research showed that this substance could create neural protection by increasing the function of the superoxide dismutase (SOD) and reducing ROS (Mehri et al., 2014). A study of linalool also showed the enzyme's ability to protect against neurotoxicity caused by ACR by increasing glutathione content and declining ROS production (Mannaa et al., 2006). Numerous studies have been performed on other antioxidant agents, including chrysin (Shukla et al., 2002), allicin (Mehri et al., 2015), and rosemary (Zhang et al., 2012), and their protective effect on ACR-induced toxicity, which supports this study. In some studies, crocetin has been used as a protective agent in neurodegenerative diseases in animal models (Ochiai et al., 2007; Waggas and Balawi, 2008). The neuroprotective effects of crocetin after seven-day administration (25, 50, and 75 μg/kg body weight) against 6-hydroxy dopamine, which causes Parkinson's disease in rats, have been reported. Reducing dopamine uptake by body tissues and thus maintaining dopamine levels in the brain was suggested as a possible mechanism (Ahmad et al., 2005). The effects of crocetin neuroprotection on brain damage in animal studies are related to the inhibition of apoptosis in the early stages of injury and its ability to promote angiogenesis in the subacute phase, which is further enhanced by the expression of vascular endothelium growth factor receptor2 (VEGFR-2) and serum response factor (SRF) (Purushothuman et al., 2013). Besides, crocetin could inhibit the death of RGC-5 cells (retinal ganglion cells) caused by H_2_O_2_ and inhibit the activity of caspase 3 and caspase-9 (Bie et al., 2011). Another study on the association between Alzheimer's and beta-amyloid has shown that crocetin, through its antioxidant effect, can prevent the fibrillation of beta-amyloid in the brain and thus prevent the progression of Alzheimer's disease (Papandreou et al., 2006). 

Some other studies show that crocetin, in addition to brain tissue, has protective effects on other organs. One of these studies was on the rate of bile flow and liver damage in rats. In this study, crocetin showed a protective effect against liver damage caused by acetaminophen and carbon tetrachloride, significantly increasing the liver's glutathione content and reducing the production of malondialdehyde. Positive and significant effects on liver tissue histopathology were also observed (Khazdair et al., 2015). Another positive effect of crocetin is its cardiovascular protection effects. For example, its antioxidant effect was studied in hypertrophy due to norepinephrine, which showed that prescribing crocetin reduced lipid peroxidase, increased glutathione content and SOD function, and improved the histopathological condition of the heart with hypertrophy (Shen and Qian, 2006). In our *in vivo* study, BMPC did not improve the behavioral index of the rats poisoned with ACR, which indicates that this substance is less effective than TSC. However, it has been shown to affect MDA and GSH but is still less effective than TSC. It seems that BMPC caused neural toxicity due to the presence of two piperazinyl groups on both sides of its structure, although this claim needs further investigation. According to the obtained results, more studies should be done to investigate the molecular mechanisms that contributed to the neuroprotective effects of TSC in *in vitro* models such as the effects of TSC on ROS level and apoptosis induced by ACR. Also, in *in vivo* model, some histopathological surveys on the brain tissue after acrylamide exposure is needed.

Oxidative stress plays a crucial role in ACR-induced neural toxicity. Cellular toxicity induced by ACR is inhibited by pretreatment with TSC and BMPC. These compounds also reduce ACR toxicity by increasing GSH content and reducing the rate of lipid peroxidation in the rat brain. In the behavioral examination, TSC was able to improve the gait disorder in the presence of ACR. Still, BPMC does not have this property in low doses and even induces toxicity in higher doses in rats. 

## Conflicts of interest

The authors have declared that there is no conflict of interest.
